# Immunomodulation of Melanoma by Chemo-Thermo-Immunotherapy Using Conjugates of Melanogenesis Substrate NPrCAP and Magnetite Nanoparticles: A Review

**DOI:** 10.3390/ijms23126457

**Published:** 2022-06-09

**Authors:** Yasuaki Tamura, Akira Ito, Kazumasa Wakamatsu, Takafumi Kamiya, Toshihiko Torigoe, Hiroyuki Honda, Toshiharu Yamashita, Hisashi Uhara, Shosuke Ito, Kowichi Jimbow

**Affiliations:** 1 Department of Molecular Therapeutics, Center for Food & Medical Innovation, Institute for the Promotion of Business-Regional Collaboration, Hokkaido University, North 8 West 5, Kita-ku, Sapporo 060-0808, Japan; ytamura@sci.hokudai.ac.jp; 2 Department of Chemical Systems Engineering, School of Engineering, Nagoya University, Furo-cho, Chikusa-ku, Nagoya 464-8603, Japan; ito.akira@material.nagoya-u.ac.jp; 3 Institute for Melanin Chemistry, Fujita Health University, 1-98 Dengakugakubo, Kutsukake-cho, Toyoake 470-1192, Japan; kwaka@fujita-hu.ac.jp (K.W.); sito@fujita-hu.ac.jp (S.I.); 4 Department of Dermatology, Sapporo Medical University School of Medicine, South 1 West 17, Cyuo-ku, Sapporo 060-8556, Japan; kamitaka@sapmed.ac.jp (T.K.); yamasita@shiroishi-hifu.jp (T.Y.); uharah@sapmed.ac.jp (H.U.); 5 Department of Pathology 1, Sapporo Medical University School of Medicine, South 1 West 17, Cyuo-ku, Sapporo 060-8556, Japan; torigoe@sapmed.ac.jp; 6 Department of Biomolecular Engineering, School of Engineering, Nagoya University, Furo-cho, Chikusa-ku, Nagoya 464-8603, Japan; honda@chembio.nagoya-u.ac.jp; 7 Institute of Dermatology & Cutaneous Sciences, 1-27, Odori West 17, Cyuo-ku, Sapporo 060-0042, Japan

**Keywords:** melanoma, chemo-thermo-immunotherapy, melanogenesis, magnetite nanoparticle, drug delivery system, heat shock protein, in situ vaccine therapy, immune checkpoint inhibitor

## Abstract

A major advance in drug discovery and targeted therapy directed at cancer cells may be achieved by the exploitation and immunomodulation of their unique biological properties. This review summarizes our efforts to develop novel chemo-thermo-immunotherapy (CTI therapy) by conjugating a melanogenesis substrate, *N*-propionyl cysteaminylphenol (NPrCAP: amine analog of tyrosine), with magnetite nanoparticles (MNP). In our approach, NPrCAP provides a unique drug delivery system (DDS) because of its selective incorporation into melanoma cells. It also functions as a melanoma-targeted therapeutic drug because of its production of highly reactive free radicals (melanoma-targeted chemotherapy). Moreover, the utilization of MNP is a platform to develop thermo-immunotherapy because of heat shock protein (HSP) expression upon heat generation in MNP by exposure to an alternating magnetic field (AMF). This comprehensive review covers experimental in vivo and in vitro mouse melanoma models and preliminary clinical trials with a limited number of advanced melanoma patients. We also discuss the future directions of CTI therapy.

## 1. Introduction

The management of advanced metastatic melanoma is an extremely difficult challenge for physicians and scientists because of the limited availability of effective therapies. There is, therefore, a critical need to develop innovative therapies for the control of advanced melanoma. The exploitation of biological properties unique to cancer cells may provide a novel approach to overcome this difficult challenge. 

The biological property unique to melanoma cells and their precursor cells, melanocytes, is the biosynthesis of melanin pigments, melanogenesis, within specific cellular compartments called melanosomes. Melanogenesis begins with the conversion of amino acid tyrosine to 3,4-dihydroxyphenylalanine (dopa) and subsequently to dopa quinone in the presence of tyrosinase. This pathway is unique to all melanocytes and melanoma cells, including “amelanotic” melanoma [1]. With the interaction of melanocyte-stimulating hormone (MSH) and the melanocortin 1 receptor (MC1R) [2], the melanogenesis cascade proceeds with activation of microphthalmia transcription factor (MITF) for the induction of either eu- or pheomelanin biosynthesis. Tyrosinase is the major player of this cascade. It is a glycoprotein and its glycosylation process is regulated by a number of molecular chaperones, including calnexin, in the endoplasmic reticulum. Vesicular transport then carries tyrosinase and its related proteins (TRPs) from the trans-Golgi network to melanosomal compartments. A significant number of transporters in this process are involved in early melanosomal maturation, to which early and late endosomes are closely associated [3]. Once melanin biosynthesis is completed, melanosomes move along dendritic processes on melanocytes and are transferred to surrounding keratinocytes in skin. However, melanoma cells do not develop many dendrites and retain melanosomes within their cytoplasm, hence forming a “black mole” [4].

**Figure 1:**
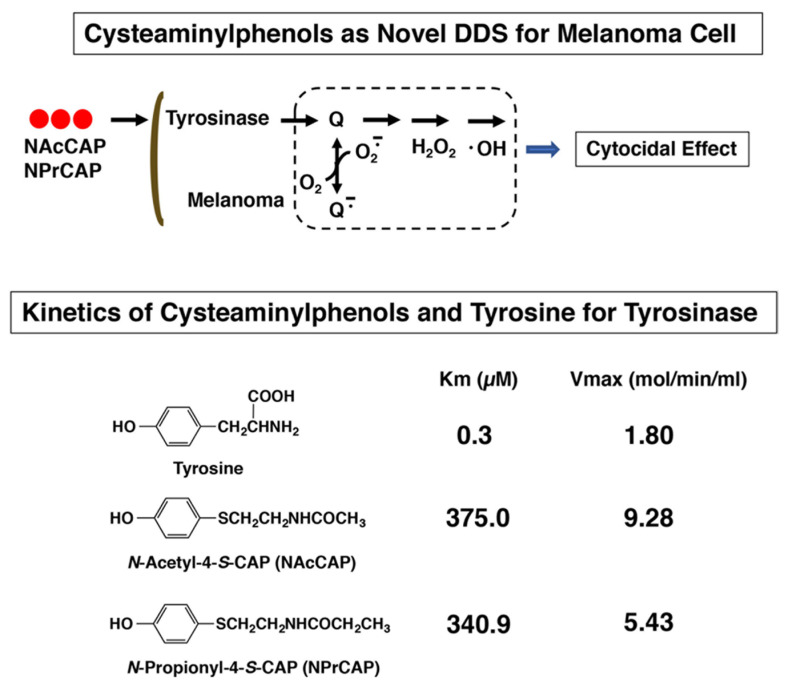
Comparison of kinetics of tyrosine, NAcCAP and NPrCAP for mushroom tyrosinase. NAcCAP and NPrCAP produce a significant amount of cytocidal free radicals through the interaction with tyrosinase.

Tyrosine analogs that are the substrates of the melanin-forming enzyme tyrosinase can be some of the best candidates for developing specific melanoma-targeting drugs and therapies. *N*-acetyl and *N*-propionyl derivatives of 4-*S*-cysteaminylphenol (NAc- and NPr-CAP) were synthesized and found to be much better substrates of tyrosinase than tyrosine (Figure 1) [5].

**Figure 2:**
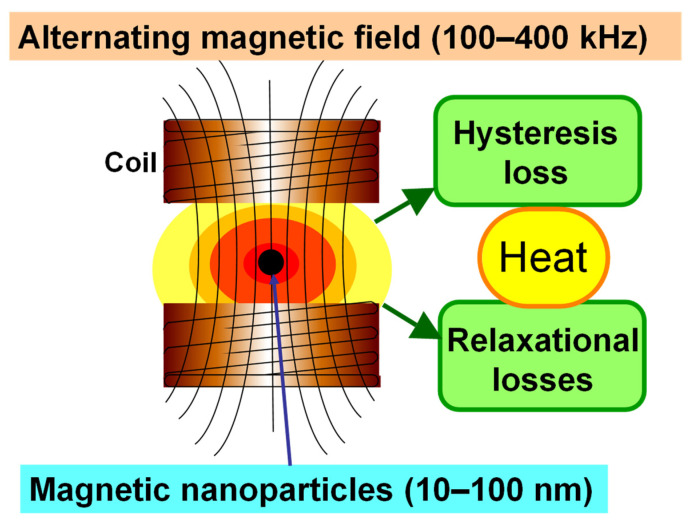
Mechanism of heat generation of magnetic nanoparticles under an alternating magnetic field.

Hyperthermia provides a promising approach for cancer treatment. In particular, photothermal therapy and magnetic hyperthermia have been extensively studied in scientific research laboratories [6]. In photothermal therapy, a laser is used to irradiate the tumor lesions, and the optical energy can be converted into heat by photothermal conversion agents such as indocyanine green [7], IR820 [8], or gold nanoparticles [9]. Intracellular hyperthermia using magnetite nanoparticles (MNP) (10–100 nm-sized, Fe_3_O_4_) has been shown to be effective in treating cancers [10,11]. Incorporated MNP generate heat within the cells after exposure to an alternating magnetic field (AMF), due to hysteresis loss [12,13,14,15] (Figure 2). Compared with other heating systems such as photothermal therapy, magnetic hyperthermia is advantageous because a magnetic field can penetrate deep into the body tissues and has almost no interactions with biological molecules. In this treatment, there is not only heat-mediated cell death but also an immune reaction due to the generation of heat shock proteins (HSPs) [16]. HSP expression induced by hyperthermia has been found to be involved in cancer immunomodulation, providing the basis for developing a novel cancer thermo-immunotherapy.

In this review, we introduce our efforts to develop novel chemo-thermo-immunotherapy (CTI therapy) by conjugating a melanogenesis substrate, NPrCAP, with MNP. In our approach, NPrCAP provides a unique DDS because of its selective incorporation into melanoma cells. It also functions as a melanoma-targeted chemotherapeutic drug because of its production of highly reactive free radicals. We compared chemo-therapeutic and thermo-therapeutic effects on primary transplanted B16 mouse melanoma cells on the flank with and without AMF exposure (heat generation) and then examined the immune-therapeutic effect on a second re-challenge transplant of the same melanoma cells on the opposite side flank to evaluate if the growth of distant metastatic melanomas can be inhibited. We also investigated the possible association of HSP production, CD8^+^ T cell activation and major histocompatibility complex (MHC) expression with rejection of the re-challenge melanoma transplants. We also introduce our preliminary therapeutic effort of this CTI strategy for a limited number of advanced melanoma patients.

## 2. Synthesis of Novel Conjugates of NPrCAP and Magnetite Nanoparticles for Developing Melanoma-Targeted Chemo-Thermo-Immunotherapy (CTI Therapy)

### 2.1. Preparation of 4-S-CAP-Loaded Magnetite Cationic L Iposomes and Measurement of In Vitro/In Vivo Antimelanoma Effects; Starting Rational Basis for Developing CTI Therapy

**Figure 3:**
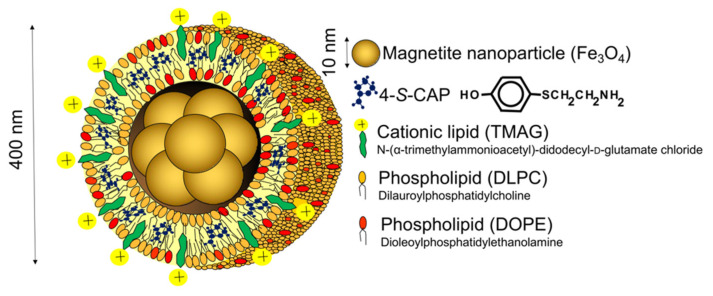
Illustration of 4-*S*-cysteaminylphenol (4-*S*-CAP)/magnetite cationic liposome (MCL).

CTI therapy is based on the combination of chemotherapy using melanogenesis substrates and hyperthermia using magnetic nanoparticles, and the resulting antitumor immune response induced by in situ vaccination through dying tumor cells. Analogs of the melanogenesis substrate tyrosine are good candidates, such as the sulfur homolog of tyrosine 4-*S*-cysteaminylphenol (4-*S*-CAP), which causes cytotoxicity of melanoma [17,18] and can be used for melanoma-targeted chemotherapy. In addition, intracellular hyperthermia can be generated by loading magnetic nanoparticles into tumor cells followed by inductive heating of the nanoparticles under an alternating magnetic field (AMF). A promising approach of the initial step to improve uptake of nanoparticles into tumor cells was to use cationic liposomes. We developed magnetite cationic liposomes (MCLs) that have 10-fold higher affinity for tumor cells than neutrally charged magnetite liposomes [19]. To test the combined effects of chemotherapy using 4-*S*-CAP and hyperthermia using MNP on melanoma, we prepared 4-*S*-CAP-loaded magnetite cationic liposomes (4-*S*-CAP/MCLs) (Figure 3) [20].

An in vitro experiment showed that 4-*S*-CAP in 4-*S*-CAP/MCLs had a dose-dependent antiproliferative effect on B16 melanoma cells, and the combination treatment of 4-*S*-CAP with hyperthermia was determined to have an additive effect [20]. As a mechanism, the cytotoxicity of 4-*S*-CAP in melanoma cells depends mostly on its production of reactive oxygen species (ROS) [21]. Hyperthermia also induces ROS in various cells [22], and ROS may play an important role in the additive effect of the combined treatment of 4-*S*-CAP and hyperthermia on melanoma cells. Moreover, 4-*S*-CAP/MCLs were injected into melanoma nodules in mice and the mice were irradiated with an AMF for 30 min. During AMF irradiation, the temperature of the melanoma nodules increased to 45 °C and tumor growth was strongly suppressed for 12 days, including complete regression of 17% (1/6) of the melanoma nodules [20]. These results suggest that melanogenesis substrate-conjugated magnetic nanoparticles are a potent tool in melanoma therapy.

### 2.2. Synthetic Method of NPrCAP-SH for CTI Therapy

A synthetic route for *N*-(1-mercaptopropionyl)-4-*S*-cysteaminyl phenol (NPrCAP-SH) has been reported already [8]. However, there was room for improvement in the synthetic process of NPrCAP-SH, because (1) the reagent *N*-succinimidyl-3-[2-pyridyldithio] propionate, used in the process described in the previous paper [8], was very expensive, and (2) it was not easy to separate NPrCAP-SH from the by-products yielded in that synthetic process. Thus, it was necessary to develop a new synthetic method that shortens the reaction time, uses less expensive reagents and can generate NPrCAP-SH with larger quantities and higher purity.

**Figure 4:**
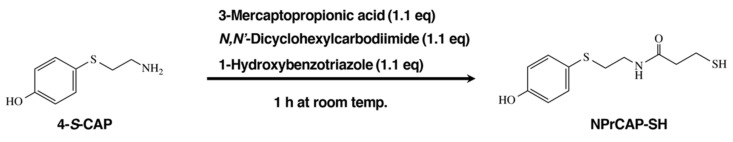
One-pot synthesis of NPrCAP-SH.

Obtained by hydrolyzing *N*-acetyl-4-*S*-CAP (NAcCAP) with 6 M HCl by the method of Padgette et al. [23], 4-*S*-CAP was reacted with 3-mercaptopropionic acid, *N*,*N*’-dicyclohexylcarbodiimide and 1-hydroxybenzotriazole in *N*,*N*-dimethylformamide for 1 h at room temperature. The resultant oily compound was purified by silica gel column chromatography to give NPrCAP-SH (90%) as a colorless crystal after recrystallization (ethyl acetate-ether). Thus, we have established an efficient and reproducible one-pot method for the synthesis of NPrCAP-SH (Figure 4).

**Figure 5:**
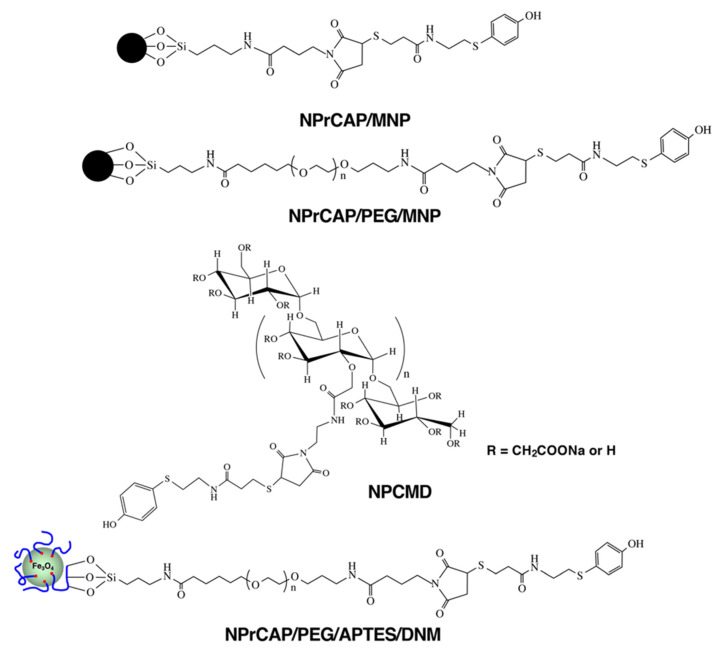
Various kinds of NPrCAP derivatives bound to MNP. NPrCAP/MNP: *N*-propionyl-4-*S*-cysteaminylphenol/magnetite nanoparticle. NPrCAP/PEG/MNP: *N*-propionyl-4-*S*-cysteaminylphenol/polyethylene glycol/magnetite nanoparticle. NPCMD: *N*-propionyl-4-*S*-cysteaminylphenol/maleimide-dextran. NPrCAP/PEG/APTES/DNM: *N*-propionyl-4-*S*-cysteaminylphenol/polyethylene glycol/3-aminopropyltriethoxysilane/dextran nanomagnetite.

We synthesized four NPrCAP derivatives bound to MNP as shown in Figure 5 for CTI therapy, and we have already reported the synthetic methods for three of these derivatives (NPrCAP/MNP, NPrCAP/PEG/MNP and NPCMD) [8,24,25,26]. In this review, we report a new method for the synthesis of NPrCAP/PEG/APTES/DNM, which is described below.

### 2.3. Synthesis of NPrCAP/PEG/APTES/DNM Bound to Dextran Nanomagnetite

**Figure 6:**
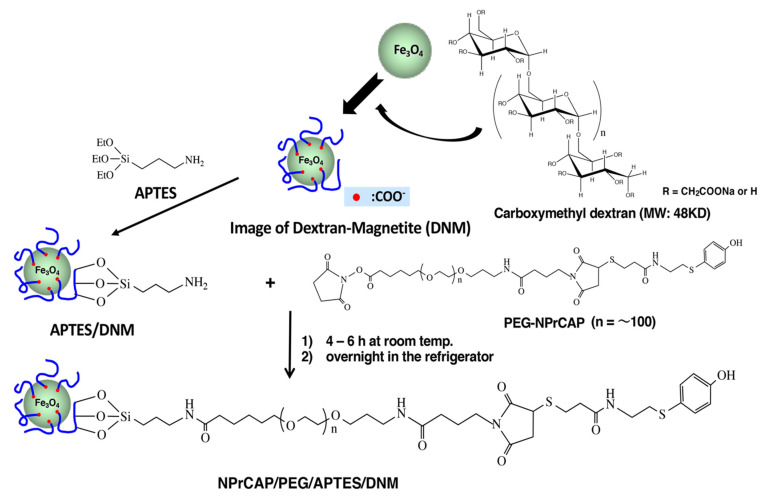
Image of DNM and synthesis of NPrCAP/PEG/APTES/DNM.

Dextran nanomagnetite (DNM) was prepared by adding dextran in water to a magnetite suspension. DNM thus prepared was first reacted with 3-aminopropyltriethoxysilane (APTES) to form APTES/DNM, and then with PEG-NPrCAP obtained by reacting NPrCAP-SH and PEG for 1 h at room temperature. The mixture was kept for 4 to 6 h at room temperature, and then kept in a refrigerator overnight to synthesize NPrCAP/PEG/APTES/DNM (Figure 6).

### 2.4. Quantification of NPrCAP Bound to DNM

**Figure 7:**
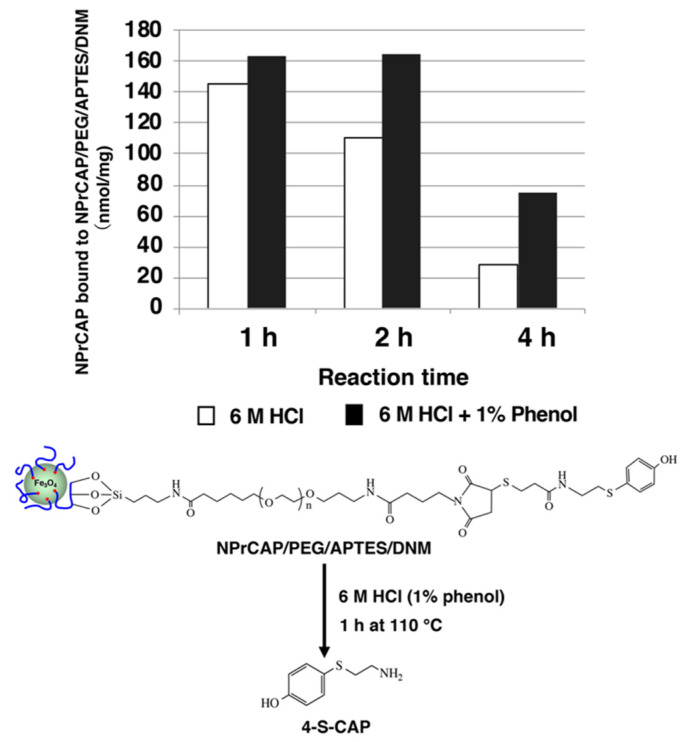
Quantification of NPrCAP bound to NPrCAP/PEG/APTES/DNM, and production of 4-*S*-CAP from NPrCAP/PEG/APTES/DNM by 6 M HCl containing 1% phenol.

In order to quantify the amount of NPrCAP bound to DNM, 6 M HCl with or without 1% phenol was added to the NPrCAP/PEG/APTES/DNM suspension and reacted for up to 4 h at 110 °C to produce 4-*S*-CAP. When hydrolyzed with 6 M HCl in the absence of phenol, the amount of 4-*S*-CAP yielded after 4 h was reduced to 20% of the amount yielded after 1 h (Figure 7). The reduction was considered to be due to the decomposition of 4-*S*-CAP during HCl hydrolysis. On the other hand, the inclusion of 1% phenol in 6 M HCl suppressed the decomposition of 4-*S*-CAP, which did not decompose even with 2 h reaction time. Based on the above results, the following experimental conditions to check the amount of NPrCAP bound to NPrCAP/PEG/APTES/DNM were established: (1) the NPrCAP/PEG/APTES/DNM suspension is reacted under 6 M HCl containing 1% phenol for 1 h, and (2) the reaction mixture is diluted 10-fold with 0.1M HCl and then 4-*S*-CAP is quantified by high-performance liquid chromatography (HPLC) analysis (Figure 7).

### 2.5. The Different Reactivities of NPrCAP/MNP and NPrCAP/PEG/APTES/DNM as Substrates for Tyrosinase

**Figure 8:**
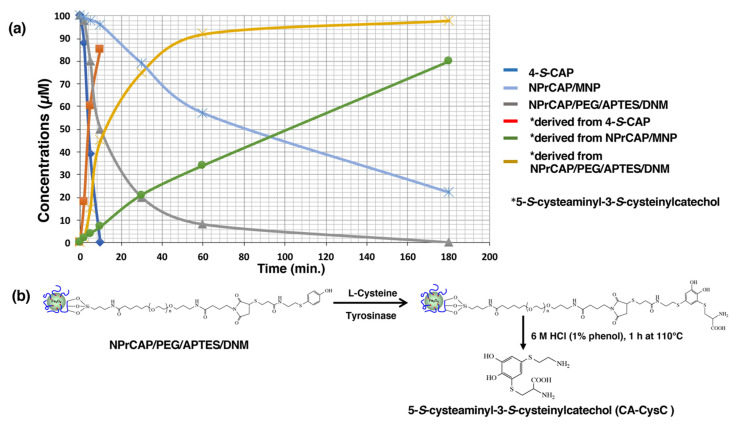
(**a**) Tyrosinase reaction of 4-*S*-CAP, NPrCAP/PEG/APTES/DNM and NPrCAP/MNP, and yield of 5-*S*-cysteaminyl-3-*S*-cysteinylcatechol (CA-CysC). NPrCAP/MNP and NPrCAP/PEG/APTES/DNM are incorporated into the tyrosinase oxidative reaction in vitro. The concentrations of the substrate remaining as 4-*S*-CAP and the CA-CysC produced were measured by HPLC analysis after hydrolysis with 6 M HCl. * is CA-CysC (derived from 4-*S*-CAP, NPrCAP/MNP and NPrCAP/PEG/APTES/DNM). (**b**) Tyrosinase oxidation of NPrCAP/PEG/APTES/DNM in the presence of cysteine followed by 6 M HCl containing 1% phenol yielded CA-CysC as well as 4-*S*-CAP and NPrCAP/MNP.

We examined whether NPrCAP/MNP and NPrCAP/PEG/APTES/DNM could act as substrates for tyrosinase; 4-*S*-CAP itself was found to be a good substrate for tyrosinase because tyrosinase oxidation of 4-*S*-CAP in the presence of cysteine yielded 5-*S*-cysteaminyl-3-*S*-cysteinylcatechol (CA-CysC) through *ortho*-quinone within 10 min (Figure 8a) [8,27,28]. HPLC analysis showed that the reaction was almost completed within 10 min, with half of the 4-*S*-CAP remaining after 4.2 min [8,27]. HPLC analysis showed that CA-CysC derived from 4-*S*-CAP was produced at 85 µM (85% yield) at 10 min. The reaction rate constant (*k*) of 4-*S*-CAP was 0.17 min^−1^. As NPrCAP/MNP has the same structural units as 4-*S*-CAP, it was expected to be a substrate for tyrosinase. If this were the case, CA-CysC would be obtained by HCl hydrolysis, in the presence of 1% phenol, of the cysteinylcatechol derivative of NPrCAP/MNP produced after tyrosinase oxidation of NPrCAP/MNP in the presence of cysteine. NPrCAP/MNP fell to half of the initial concentration after 82 min, and CA-CysC produced after 180 min was 80 µM (80% yield) (Figure 8a). Thus, the ratio of 4-*S*-CAP to NPrCAP/MNP in the reaction velocity on tyrosinase oxidation was 19.5, and the reaction rate constant (*k*) of NPrCAP/MNP was 8.5 × 10^−3^ min^−1^. These results indicate that NPrCAP/MNP served as a substrate for tyrosinase. On the other hand, in the case of NPrCAP/PEG/APTES/DNM, the time for reduction to half of the initial concentration was 17 min, and the concentration of CA-CysC produced after 180 min was 98 µM (98% yield) (Figure 8a,b). The ratio of 4-*S*-CAP to NPrCAP/PEG/APTES/DNM in the reaction velocity on tyrosinase oxidation was 4.0, and the reaction rate constant (*k*) of NPrCAP/PEG/APTES/DNM was 4.1 × 10^−2^ min^−1^. Thus, the tyrosinase oxidation of NPrCAP/PEG/APTES/DNM was about five times faster than that of NPrCAP/MNP. This was predicted because (1) the dispersibility of NPrCAP/PEG/APTES/DNM is greater and (2) in NPrCAP/PEG/APTES/DNM, the side chain is longer, and the steric hindrance of the aromatic ring site was alleviated.

## 3. Selective Inhibition of Melanoma Growth by NPrCAP/MNP Conjugates in a Mouse Melanoma Model

### 3.1. Melanoma-Targeting Drug Delivery and Growth Inhibition

**Figure 9:**
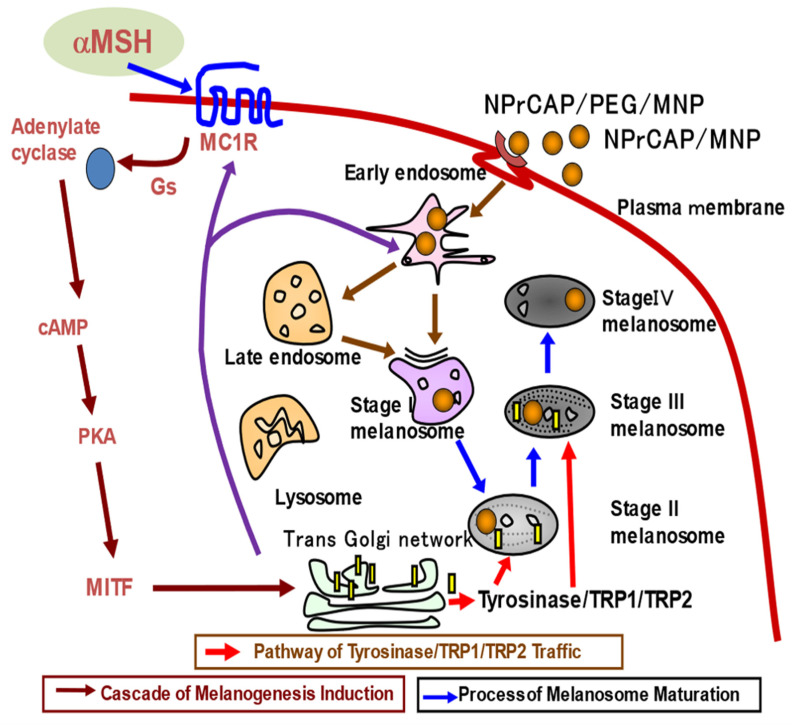
Selective accumulation of NPrCAP/MNP and NPrCAP/PEG/MNP.

Two basic findings emerged in our studies exploiting the melanogenesis cascade to develop a better, novel therapeutic approach to melanoma treatment. One is that modified tyrosinase substrates such as the sulfur homologue of tyrosine, cysteinylphenol (CP), and its amine analogue cysteaminylphenol (CAP), will be selectively incorporated into melanoma cells through active transport on the cell surface, which we believe can be used as the basis for developing a novel DDS (Figure 9) [29,30].

Another finding is that melanin biosynthesis per se, if overproduced, is toxic to melanoma cells through the production of quinone and cytotoxic free radicals, which can be used as a potential source for the development of pharmacologic and immunologic anti-melanoma agents [31].

**Figure 10:**
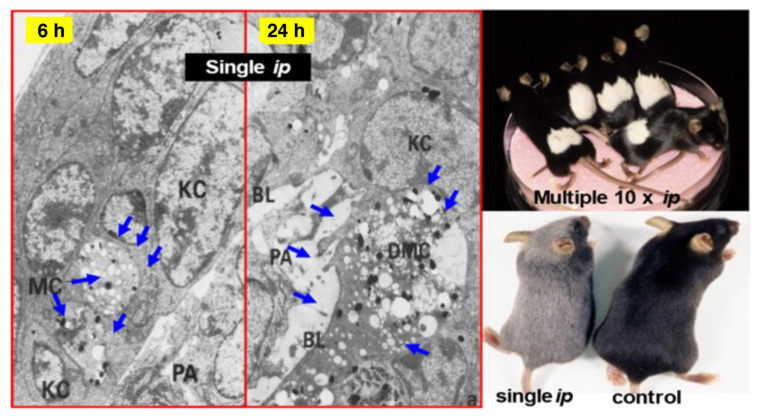
Selective cytotoxicities of NAcCAP and NPrCAP to black hair follicles of newborn and adult C57BL/6 mice by *ip* administration. (KC: keratinocyte; MC: melanocyte; DMC: degenerating melanocyte; BL: basal lamina; PA: hair papilla). Blue arrows indicate the vacuolar degeneration in melanocytes.

The melanoma-targeting DDS and selective cytotoxic properties were shown by a number of approaches. For example, both NPrCAP and NAcCAP can selectively disintegrate follicular melanocytes after single or multiple *ip* administration to new-born or adult C57BL/6 black mice. At a site on adult mice where hair follicles were plucked to stimulate new melanocyte growth and activate tyrosinase biosynthesis, repeated *ip* administration of NPrCAP yielded white follicles with 100% success. A single *ip* injection of NPrCAP into newborn mice resulted in the development of silver follicles in the entire body coat. The selective disintegration of melanocytes can be seen by electron microscopic examination as early as 12 h after a single *ip* administration. None of surrounding keratinocytes or fibroblasts showed membrane degeneration or cell death (Figure 10) [32,33]. In experiments using melanoma-bearing mice, NAcCAP and NPrCAP were found to be selectively incorporated into melanoma transplants and retained within melanoma cells, exerting a cytotoxic effect through oxidative stress that may derive from the tyrosinase-catalyzed production of cytotoxic free radicals from the tyrosine analogs (chemotherapeutic effect) [34].

**Figure 11:**
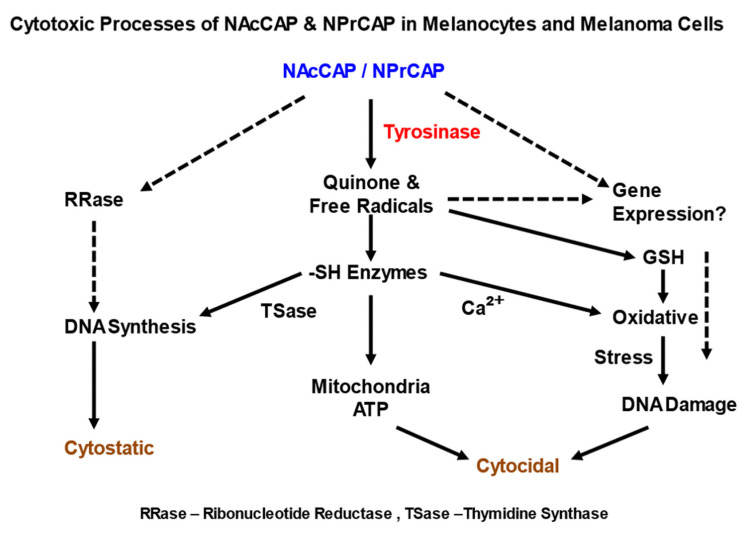
Molecular cascade of cytotoxic action of NAcCAP and NPrCAP in melanocytes and melanoma cells.

The specific cytotoxicities of NPrCAP and NAcCAP were further examined in various types of cultured cells by 3-[4,5-dimethylthiazol-2-yl]-2,5-diphenyltetrazolium bromide (MTT) assay [35,36]. Among them, only melanocytic cells and HeLa cells showed low IC50 values. The administration of high concentrations caused irreversible damage to melanoma cells in colony formation assays and the cytotoxicity to these cells was dose-dependent. However, the cytotoxicity to HeLa cells in DNA synthesis was transient and reversible. The molecular mechanism for the cytotoxic action by NAcCAP and NPrCAP appears to involve two major biological processes. One is cytostatic action that derives from DNA synthesis inhibition through the interaction of quinone and free radicals with the SH-enzyme protein disulphide isomerase. Another is cytocidal action by damage of DNA and mitochondrial ATP through oxidative stress. Combining NAcCAP with buthionine sulfoxide (BSO), which blocks the effect of antioxidants, revealed a marked growth inhibition of cultured melanoma cells, indicating again that the selective cytotoxicity of our CAP is related to quinone and free radicals (Figure 11) [36].

In B16F10 melanoma-bearing mice, the administration of NAcCAP, when combined with BSO, yielded marked growth inhibition of melanoma cells, indicating that the selective cytotoxicity of our CAP is related to the production of quinones and free radicals. In vivo lung metastasis assays in these mice showed a decreased number of lung melanoma colonies in the presence of BSO [32]. However, the problem with NAcCAP administration was that a fairly large number of amelanotic melanoma lesions was seen to grow in the lung. Administration of NPrCAP, however, has improved this “amelanosis” problem, showing fewer amelanotic lesions and increased cytotoxic growth inhibition. 

Subsequent experiments using NPrCAP conjugated with MNP revealed selective disintegration of melanoma tissues upon exposure to AMF [9]. Thus, our approach of exploitation of melanogenesis substrates and synthesis of NPrCAP has provided a firm basis to develop melanoma-targeting rational therapy. In addition, NAcCAP may be an ideal choice for the development of a novel depigmenting agent for skin diseases such as melasma [5].

### 3.2. Growth Inhibition of Re-Challenge Melanoma Transplant

**Figure 12:**
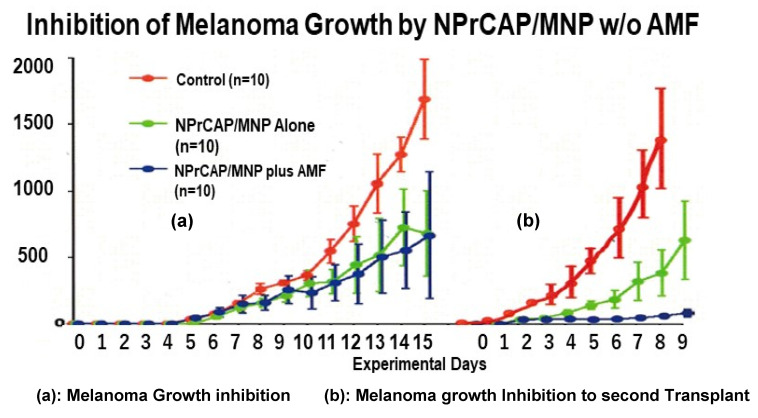
Melanoma growth inhibition by NPrCAP/MNP with/without AMF. (**a**) Growth of primary melanoma transplants in mice. (**b**) Growth of second re-challenge melanoma tumors transplanted on the flank opposite from the primary tumor on day 53, 40 days after excision of the primary tumors.

We first evaluated the chemotherapeutic effect of NPrCAP/MNP on primary transplants of B16F1 melanoma with (group 1) and without (group 2) heat exposure, followed by excision of the primary tumors and re-challenge with a second B16F1 melanoma transplant. Significant growth inhibition of the primary transplants was observed in both groups of mice (Figure 12a). However, only the group of mice treated with NPrCAP/MNP plus AMF showed almost complete growth inhibition of the second melanoma transplants (Figure 12b), indicating that while NPrCAP/MNP alone had a moderate chemotherapeutic effect on the second melanoma transplants, only the group treated with both NPrCAP/MNP and AMF heat had almost complete growth inhibition of the re-challenge melanoma transplants.

**Figure 13:**
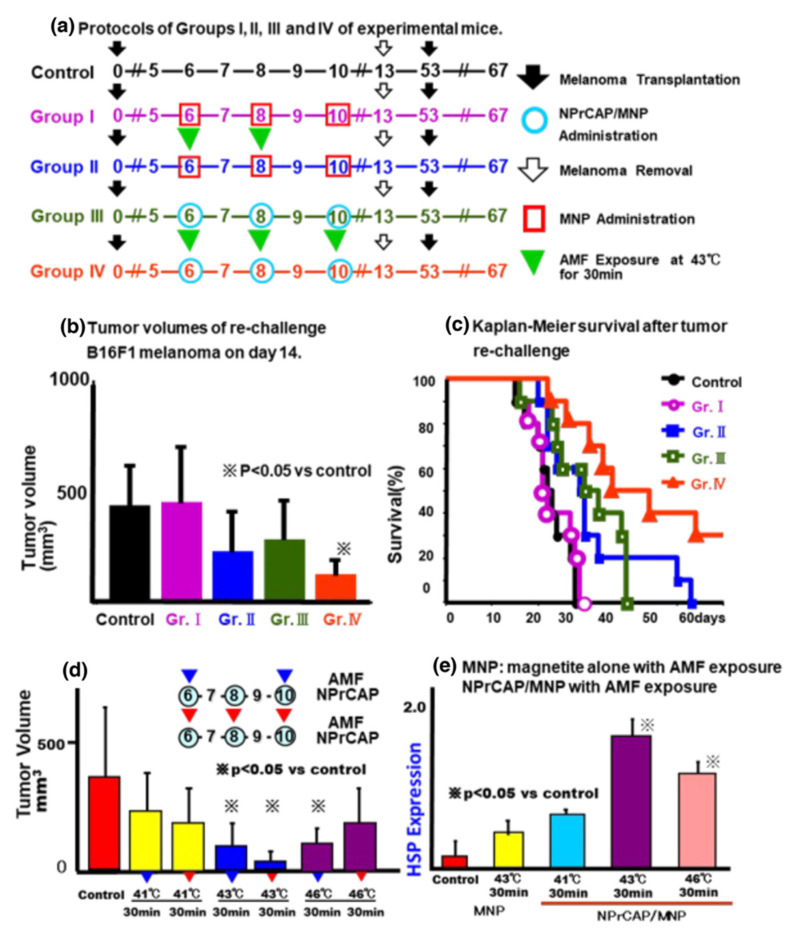
Tumor growth and survival of mice re-challenged with melanoma transplants after treatment of initial transplants; heat shock protein (HSP) expression in initial transplants. (**a**) Experimental protocols for the five groups of mice, all of which underwent initial B16F1 melanoma transplants on day 0, removal of the tumors on day 13, and re-challenge with B16F1 on the opposite flank on day 53. The Control group received no treatment, Group I received MNP without AMF on the days indicated, and Group II received MNP + AMF. Group III received NPrCAP/MNP without AMF, and Group IV received NPrCAP/MNP + AMF. (**b**) Volumes of re-challenge B16F1 tumors 14 days after transplantation (67 days after primary transplantation). (**c**) Kaplan–Meier survival curves after primary and re-challenge melanoma transplants. (**d**) Effects of AMF frequency and temperature on re-challenge B16F1 tumor volumes in mice treated with NPrCAP/MNP on days 6, 8 and 10 after primary tumor transplantation plus AMF on days 6 and 10 (blue inverted triangles) or on days 6, 8 and 10 (red inverted triangles). Tumor volumes were measured on day 67, 14 days after re-challenge transplantation. (**e**) HSP expression in primary B16F1 tumors treated with MNP or NPrCAP/MNP together with exposure to AMF at the indicated temperatures. ※ shows the significant differences between the control.

Further evaluation of re-challenge B16F1 transplants after the treatment of primary transplants was performed revealed that AMF exposure improved the tumor suppressive activity of either MNP or NPrCAP/MNP treatment, with the greatest tumor growth inhibition and survival provided by NPrCAP/MNP with AMF exposure (Figure 13) [8,9]. Our experiments indicated that the most effective primary tumor therapy for inhibiting the growth of re-challenge B16F1 melanoma transplants was obtained when the treatment was repeated three times, once every other day, with AMF exposure at a temperature of 43 °C for 30 min (Figure 13d). Moreover, NPrCAP/MNP treatment with AMF exposure resulted in a significant increase in HSP expression (Figure 13e), indicating that NPrCAP/MNP with heat exerts a thermo-immunotherapeutic effect. A previous study using cationic magneto-liposomes-mediated hyperthermia with B16 melanoma showed that hyperthermia at 46 °C once or twice led to regression of 40–90% of primary tumors and to 30–60% survival of mice, whereas hyperthermia at 43 °C failed to induce regression of the secondary tumors, with 0% survival of mice [20].

### 3.3. Induction of NPrCAP-Mediated Melanoma Apoptosis

The chemical agent NPrCAP has been shown to be a good substrate for tyrosinase and to be selectively incorporated into melanoma cells, causing cytotoxicity [27,36]. We examined the molecular mechanism of NPrCAP-mediated cytotoxicity to melanoma cells by focusing on intracellular reactive oxygen species (ROS).

When melanocytic and non-melanocytic cells were exposed to NPrCAP (0.5–3.0 mM) for 1 h and cultured for 24 more h, the growth of pigmented B16F1, 70W and M1 melanoma cells was inhibited in a concentration-dependent manner, while the growth of NIH3T3 fibroblast cells was not affected. An inactive form, *N*-propionyl-2-*S*-cysteaminylphenol, showed no growth inhibitory effect on B16F1 or NIH3T3 cells. 

To examine the mechanism of the cell death induced by NPrCAP, cellular DNA and caspase activation were analyzed by flow cytometry and caspase 3 assay. After pigmented B16F1, non-pigmented TXM18 melanoma, NIH3T3 fibroblast and RMA mouse lymphoma cells were exposed to 1 mM NPrCAP for 1 h and cultured for 24 more h, and adherent and floating cells were collected and processed for flow cytometry. The results showed that the sub-G1 fraction was increased in the NPrCAP-treated B16F1 cells, comparable to the increase in TNF-related apoptosis-inducing ligand (TRAIL)/Apo2L-exposed B16F1, but was not elevated in the NPrCAP-treated NIH3T3, TXM18, or RMA cells [10]. For analysis of caspase 3 activation, cells were cultured in medium containing NPrCAP for 1 h and then caspase 3/7 activity was measured using a Caspase-Glo3/7 Assay kit (Promega, Madison, WI) and a luminescence microplate reader. The assay detected caspase 3/7 activity that was remarkably increased (35.8-fold) in the NPrCAP-treated B16F1 cells, comparable to TRAIL-exposed cells. On the other hand, NIH3T3, RMA and TXM18 cells treated with NPrCAP showed increases of only 4.1-, 1.4- and 1.4-fold, respectively [10]. These findings suggested that NPrCAP induces apoptotic cell death selectively in pigmented melanoma cells in association with increased caspase 3 activity.

**Figure 14:**
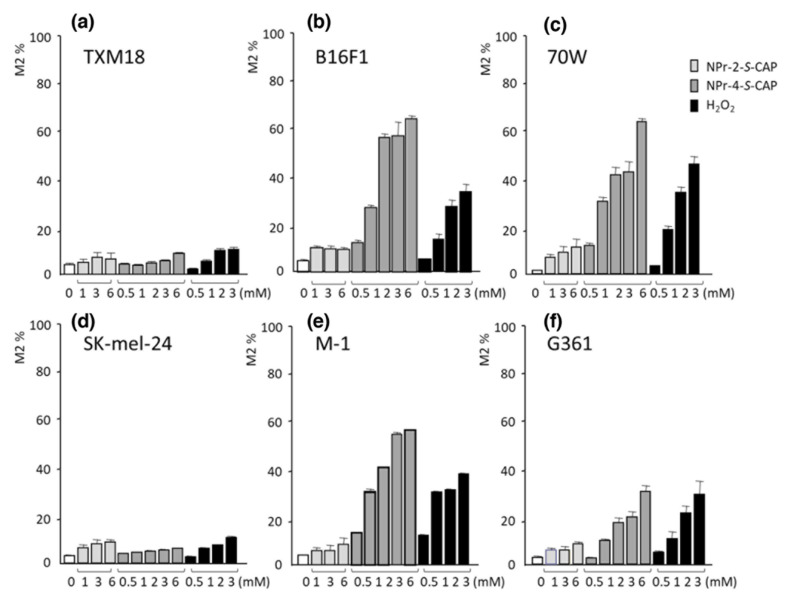
Pigmented but not non-pigmented melanoma cells produce ROS in the presence of NPr-4-*S*-CAP.

To analyze the relationship between NPrCAP-mediated apoptosis and ROS production in melanocytic cells, we examined ROS generation using flow cytometry. Pigmented and non-pigmented melanoma cells were cultured with the general ROS indicator CM-H2DCFDA (5 μM) for 30 min and then with NPrCAP (NPr-4-*S*-CAP) or the inactive NPr-2-*S*-CAP (0.5–6 mM) for 1 h. The cells were then collected and processed for flow cytometry to quantify the ROS-containing cell fraction M2 [10]. Pigmented B16F1, 70W, G361 and M-1 melanoma cells, but not non-pigmented TXM18 and SK-mel-24 melanoma cells, produced significant amounts of ROS in the presence of NPr-4-*S*-CAP (Figure 14). The results suggested that NPrCAP selectively produced ROS in pigmented melanoma cells and that melanin biosynthesis was essential for NPrCAP to produce ROS.

## 4. Specificity and Mechanism of Immunomodulation by CTI Therapy in Melanoma 

### 4.1. NPrCAP as Neo-Antigen Producer

NPrCAP is a good substrate for tyrosinase [35] and is selectively incorporated into melanoma cells, which causes cytotoxicity in vitro and in vivo [35,36]. To clarify the molecular mechanism of NPrCAP-mediated cytotoxicity to melanoma cells, Ishi-Osai reported that mice treated with intratumoral injections of NPrCAP to suppress the growth of primary B16F1 melanoma transplants also rejected secondary re-challenge tumors [10]. The participation of CD8^+^ T cells was suggested for the NPrCAP-mediated anti-B16F1 melanoma immunity.

Phenolic substrates as prohaptens are oxidized by tyrosinase to produce *ortho*-quinones, which act as haptens that covalently bind to tyrosinase or other melanosomal proteins to generate potential neo-antigens [37,38,39]. These neo-antigens trigger an immunological response cascade that results in a melanocyte-specific, delayed-type hypersensitivity reaction leading to melanocyte elimination or melanoma rejection. 

**Figure 15:**
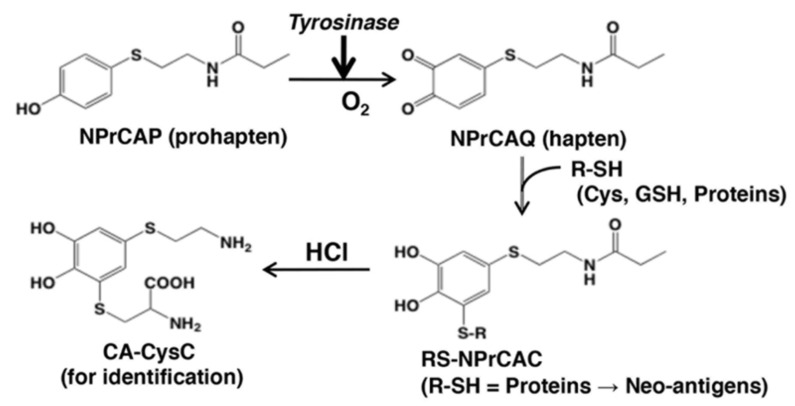
Tyrosinase activation of NPrCAP (prohapten) and binding of the quinone-hapten NPrCAQ to proteins through cysteine residues [28]. Oxidation of NPrCAP with tyrosinase produces the quinone NPrCAQ, which binds to thiols (cysteine, reduced glutathione, melanosomal proteins). The production of NPrCAQ-thiol adducts can be confirmed by the detection of CA-CysC after acid hydrolysis.

Based on the haptenation theory, Ito et al. examined the oxidation of NPrCAP and its subsequent binding to sulfhydryl compounds (thiols) [28]. They demonstrated that NPrCAP is oxidized by tyrosinase to form a highly reactive *ortho*-quinone (*N*-propionyl-4-*S*-cysteaminyl-1,2-benzoquinone, NPrCAQ; Figure 15), which binds covalently to biologically relevant thiols, including proteins through cysteine residues. The production and release of NPrCAQ-protein adducts was verified in B16F1 melanoma cells in vitro and in B16F1 melanoma-bearing mice in vivo through the detection of CA-CysC after acid hydrolysis of the protein fraction (Figure 15). These results suggested that the phenol NPrCAP, acting as a prohapten, can be oxidized in melanoma cells by tyrosinase to the active quinone-hapten NPrCAQ, which binds to melanosomal proteins through their cysteine residues to form possible neo-antigens, thus triggering the immunological response. 

### 4.2. T-Cell Receptor Repertoires of Tumor-Infiltrating Lymphocytes

Cytotoxic T lymphocytes (CTLs) play a significant role in antitumor immunity, and the presence of tumor-infiltrating lymphocytes (TILs) has been considered to be a favorable clinical prognostic indicator [40]. To further understand the T-cell response to melanoma in CTI therapy and to develop a more effective strategy based on immunomodulation, we investigated the diversity of TILs after CTI therapy [16]. The immune response of CTLs is mediated via T-cell receptors (TCRs) consisting of α and β chains. In the variable (V) regions, the gene sequence encoding the third complementarity-determining region (CDR3), which is called the hypervariable region, is considered to play the most important role in antigen recognition [41]. We analyzed the diversity of the TCR Vβ family to investigate the qualitative changes of TILs after CTI therapy. Almost all TCR Vβ families (in total, 21 TCR Vβ families were analyzed) were detected in untreated B16 melanoma in C57BL/6 mice, whereas the TCR repertoire was restricted to a few TCR Vβ families in TILs after CTI therapy. Among them, expression of the Vβ gene was confirmed with good reproducibility, suggesting that T cells expressing the TCR Vβ were activated by CTI therapy in B16 melanoma. In addition, we succeeded in analysis of the CDR3 gene sequence of TCR Vβ in TILs after CTI therapy [28]. Consistent with our result, it was reported that a B16 melanoma-specific CD8+ T cell line, AB1, expressed TCR Vβ11 [42], suggesting that clonal expansion of Vβ11+ TILs can be a useful biomarker for the T-cell response to B16 melanoma in mice. Furthermore, the same group reported that the AB1 cells recognized a melanoma antigen, tyrosinase related protein-2 (TRP-2) peptide, which was consistent with a report by Singh et al. showing that a TRP-2 peptide-specific CD8+ T cell clone expressed Vβ11 [43]. In order to identify the antigen specificity of TILs after CTI therapy of B16 melanoma, we investigated the interferon (IFN)-γ production ability using melanoma antigen peptides such as TRP-1_222–229_, TRP-2_180–188_ and gp100_25–33_. When stimulated with the TRP-2 peptide, T cells were activated to secrete IFN-γ, indicating that TILs induced by CTI therapy of B16 melanoma responded to the TRP-2 peptide. Taken together, these findings show that tumor-specific TILs were produced after CTI therapy and suggest that TCR Vβ11+ T cells are particularly important for immunity against B16 melanoma. Moreover, the melanoma antigen peptides selected by TIL analysis (e.g., TRP-2 peptide for B16 melanoma) may be used to boost antitumor immunity induced by CTI therapy.

### 4.3. CTI Therapy as In Situ Peptide Vaccine Immunotherapy

By comparing the antitumor effect of NPrCAP/MNP with and without AMF exposure, we observed that NPrCAP/MNP with AMF exposure had a superior antitumor effect compared with that of NPrCAP/MNP alone. Furthermore, mice bearing primary melanoma tumors treated with NPrCAP/MNP plus AMF showed significant suppression of re-challenge second transplant melanoma growth, whereas NPrCAP/MNP without AMF was much less effective, with 30–50% rejection of re-challenge melanoma. These results indicate that NPrCAP/MNP with AMF exposure has a strong immunotherapeutic effect [8,9]. Therefore, we investigated the underlying mechanisms for the induction of antitumor immunity induced by NPrCAP/MNP with AMF exposure. Incorporated MNP exposed to an AMF generate heat within cells due to hysteresis loss or relaxational loss [19]. It has been demonstrated that intracellular hyperthermia using MNP is effective for the treatment of certain types of cancer, in not only primary but also metastatic lesions [44,45,46,47]. Hyperthermic treatment using cationic magnetite liposomes containing 10 nm MNP induced antitumor immunity by the enhancement of HSP expression [46]. It has been demonstrated that various types of HSPs bind antigenic peptides, and these antigen peptides are cross-presented to specific cytotoxic T cells by professional antigen-presenting cells, including dendritic cells (DCs). This exogenous pathway is called cross-presentation and is important for the development of CD8^+^ T cell responses against tumors and infectious pathogens that do not have access to the classical MHC class I pathway [48,49]. In our study using B16-OVA melanoma cells, treatment with NPrCAP/MNP with AMF exposure resulted in the increased expression of HSPs, including Hsp72, Hsp90 and ER-resident stress proteins such as gp96, in melanoma cells [11]. Moreover, these HSPs (Hsp72, Hsp90 and gp96) were secreted in extracellular milieu and were taken up by DCs. These DCs presented melanoma-associated antigen peptides (OVA peptide and TRP2 peptide) through cross-presentation of HSP-bound peptide(s) to specific CD8^+^ T cells. Among HSPs, Hsp72 was shown to be largely responsible for the augmented antigen presentation to CD8^+^ T cells. As Hsp72 is known to be most highly upregulated among several HSPs in response to heat shock, newly synthesized Hsp72 has more chances to bind melanoma-associated antigen peptides. 

**Figure 16:**
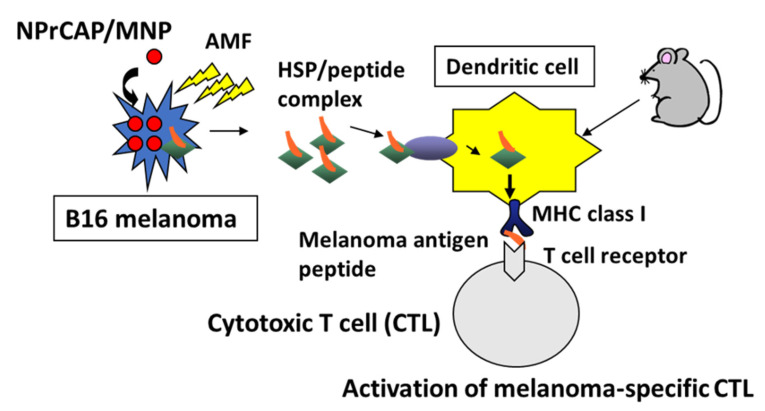
Induction of cytotoxic T cells specific for melanoma-associated antigen peptide by intracellular hyperthermia generated by NPrCAP with AMF. (1) Treatment of melanoma using NPrCAP/MNP with AMF induces various types of HSPs that bind melanoma-associated antigen peptides, and necrotic melanoma cells release HSP-antigen peptide complexes in the tumor microenvironment. (2) Infiltrated dendritic cells (DCs), which sense the inflammation induced by hyperthermia, take up HSP-peptide complexes and cross-present HSP-chaperoned antigen peptides to antigen specific cytotoxic T lymphocytes (CTLs).

Thus, our hyperthermia using NPrCAP/MNP with AMF exposure induced an anti-melanoma cytotoxic T lymphocyte (CTL) response through cross-presentation of melanoma-specific antigen peptides bound to hyperthermia-induced HSPs by DCs. More importantly, intracellular hyperthermia using NPrCAP/MNP can be a promising treatment for the prevention of recurrence and/or distant metastasis of melanoma, because systemic antimelanoma immunity is induced by this therapy (Figure 16).

If the treatment with NPrCAP/MNP plus AMF could prevent distant melanoma metastasis such as lung and distant cutaneous metastases, it would be a great boon for patients with advanced melanoma. Therefore, we examined whether treatment of primary cutaneous B16 melanoma with intracellular hyperthermia using NPrCAP/MNP with AMF can inhibit lung colonization of intravenously injected secondary challenge B16 melanoma cells. We observed that NPrCAP/MNP plus AMF clearly inhibited lung metastasis compared with NPrCAP/MNP alone. These results indicated that intracellular hyperthermia using NPrCAP/MNP with AMF elicited systemic antimelanoma immunity and prevented lung metastasis and the recurrence of melanoma.

Thus, CTI therapy using NPrCAP/MNP with AMF against advanced melanoma is a promising strategy not only for the treatment of primary melanoma but also for prevention of the recurrence of melanoma.

## 5. Approach to Advanced Melanoma Patients

### 5.1. Scale-Up Production of NPrCAP/PEG/APTES/DNM for Clinical Application

Based on NPrCAP/PEG/APTES/DNM, which is a PEG-mediated conjugate of NPrCAP and APTES with DNM, we tested further improvements of the synthesis conditions for the development of a good manufacturing practice (GMP)-based production process. Water dispersibility is important for injectable drugs. We found that the aggregation of NPrCAP/PEG/APTES/DNM was caused by particle-to-particle interactions due to APTES. By reducing the iron concentration from 10 mg/mL to 1 mg/mL during the APTES reaction, we found that the particle size of NPrCAP/PEG/APTES/DNM did not increase even after the reaction. Thus, this new formulation of NPrCAP/PEG/APTES/DNM can pass through a 0.2 µm filter, enabling sterilization, which is extremely important in the manufacture of drugs. Furthermore, the equipment for the production of NPrCAP/PEG/APTES/DNM, compliant with GMP, was installed in the laboratory of Meito Sangyo Co., Ltd. (Nagoya, Japan). Meito Sangyo has manufactured ferucarbotran, the drug substance of Resovist [50], which is sold by Bayer Schering Pharma (Berlin, Germany) as a clinically available magnetic resonance imaging contrast agent, in compliance with GMP. As a result of repeated synthesis while complying with the standard operating procedure, a total of 14 lots (800 mL) of NPrCAP/PEG/APTES/DNM were synthesized, and the reproducibility was confirmed. Taken together, a standard for the formulation of NPrCAP/PEG/APTES/DNM was determined.

### 5.2. Preliminary Human Clinical Trial of CTI Therapy for Advanced Melanoma Patients

Based on our animal experiments and the successful production of GMP grade NPrCAP/PEG/APTES/DNM, a preliminary human clinical trial (Phase I/II) has been carried out with a limited number of stage III and IV melanoma patients, after receiving informed consents from the patients and institutional approval of our human clinical trial protocol (Clinical Trial Research No. 18-67, Sapporo Medical University). 

**Figure 17:**
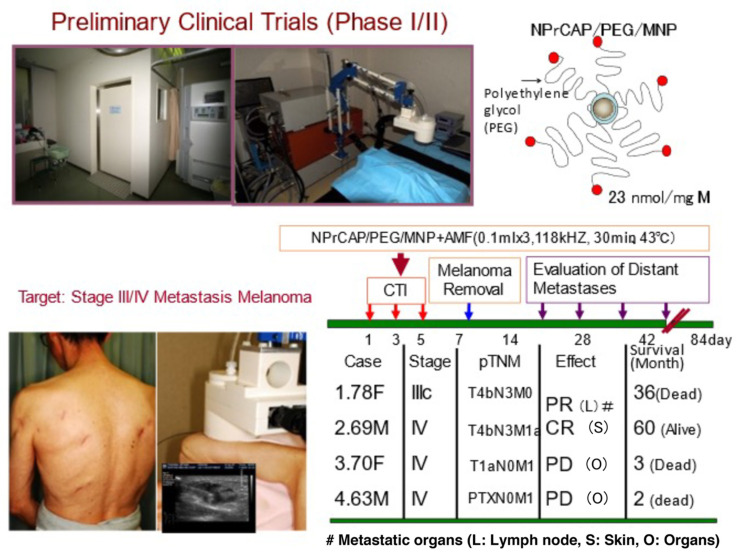
Preliminary clinical trials of CTI therapy for advanced metastatic melanoma patients in stages III and IV. Upper photos show the treatment room (**left**) and medical device for AMF exposure (**right**) for CTI therapy. The black and white ultrasound insert in the lower photo shows the drug is being given into the metastatic lymph node through an ultrasound-guided needle. # shows metastatic organs. PR: partial response, CR: complete response, PD: progressive disease.

**Figure 18:**
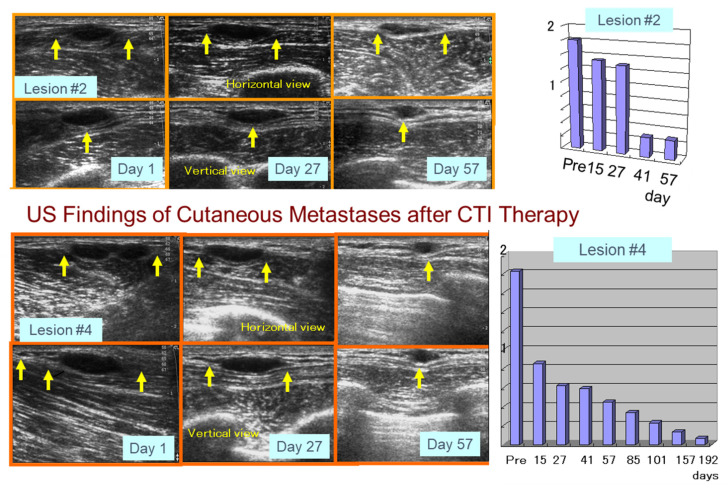
Ultrasound (US) findings of distant melanoma metastases after CTI therapy. Arrows in the photo indicate horizontal and vertical views of metastatic tumors comparing the decreased size of the tumor volume.

The therapeutic protocol basically followed an identical experimental schedule as that of the animal experiments (Figure 17 and Figure 18). Among four patients treated with NPrCAP/PEG/APTES/DNM plus AMF exposure, two of them showed complete and partial responses, respectively, and have been able to carry out normal daily activities after the CTI therapy. In one of those two responding patients, four distant cutaneous metastasis sites were evaluated and either significant regression or shrinkage of all four lesions was seen. That patient was able to survive 36 months after several cycles of CTI therapy. The pathological and immunological specimens revealed dense aggregations of lymphocytes and macrophages at the site of CTI therapy. Importantly, there was a trend toward an almost identical distribution of CD8^+^ T cells and MHC class 1 positive cells. The other responding patient had many lymph node metastases, but has survived more than 32 months so far. In order to evaluate the overall therapeutic value for advanced melanoma, it is important to have larger-scaled clinical trials and to define concisely the molecular interactions between the chemotherapeutic and thermo-immunotherapeutic effects in our CTI therapy.

## 6. Summary and Perspective

While there has been a significant advance in early detection, malignant melanoma continues to be a large contributor to cutaneous cancer-related mortality. In melanoma patients with metastases, the clinical effects of cytotoxic anticancer drugs, including decarbonize, have been limited. Over the last decade, however, an influx of novel systemic-targeted therapeutics using immune checkpoint inhibitors (ICIs) and BRAF/MEK inhibitors has been introduced and has improved both quality of life and survival for advanced stage patients. 

The approved ICIs for melanoma are the antibodies in programmed cell death protein 1 (nivolumab, pembrolizumab), cytotoxic T lymphocyte-associated antigen 4 (ipilimumab) and lymphocyte-activation gene 3 (relatlimab). They are used for the management of unresectable or metastatic melanoma, or in an adjuvant setting, regardless of BRAF-mutation status [51]. They have contributed to extending overall survival; however, the clinical effect is not fully satisfactory, particularly in acral and mucosal melanoma [52]. Furthermore, the ICIs are commonly associated with adverse events including fatigue and diarrhea from enterocolitis. Inflammatory hepatitis and inflammatory skin rashes are also common.

Targeted therapies are another approach to inhibiting uncontrolled cellular proliferation and subsequent resistance. They include BRAF/mitogen-activated protein kinase/extracellular receptor kinase (MEK) inhibition, which is seen as first-line management in patients suffering from stage III or metastatic disease with a proven mutation. Adverse events are also associated with monotherapy of these agents, such as pyrexia, fatigue, gastrointestinal upset, arthralgias, cardiovascular toxicities and epidermal neoplasms. The incidence of adverse events is, however, decreased in combination therapies. 

The long-term effects of the currently available ICIs and targeted therapies are limited. Therefore, it is necessary to apply further efforts in developing melanoma-targeted immunomodulative systemic therapies. In this review, we have introduced our approach that produces chemotherapeutic, thermotherapeutic and immunotherapeutic effects. This approach is based upon the following discoveries:

(1) Treatment of primary melanoma transplants with conjugates of the melanogenesis substrate NPrCAP and MNPs (NPrCAP/MNPs), together with AMF exposure, can result in the generation of cytotoxic T cells that inhibit the growth of re-challenge melanomas that are transplanted at the opposite site of the body from the primary tumors;
(2) NPrCAP alone appears to generate some chemotherapeutic and immunotherapeutic properties through both apoptotic and non-apoptotic processes;
(3) A unique melanogenesis cascade can be employed for developing a novel targeted chemo-thermo-immunologic strategy (CTI Therapy) for advanced melanoma patients. It is achieved by conjugating NPrCAP with magnetite nanoparticles (NPrCAP/MNPs) and, together with AMF exposure, NPrCAP/MNPs can induce cytotoxic T cells that inhibit the growth of re-challenge melanoma transplants at the opposite side of the body from the treated primary melanoma.

It may, therefore, be considered that we have provided evidence that the unique melanogenesis cascade can be employed for developing a novel chemo-thermo-immunologic strategy (CTI therapy) for advanced melanoma patients.

**Figure 19:**
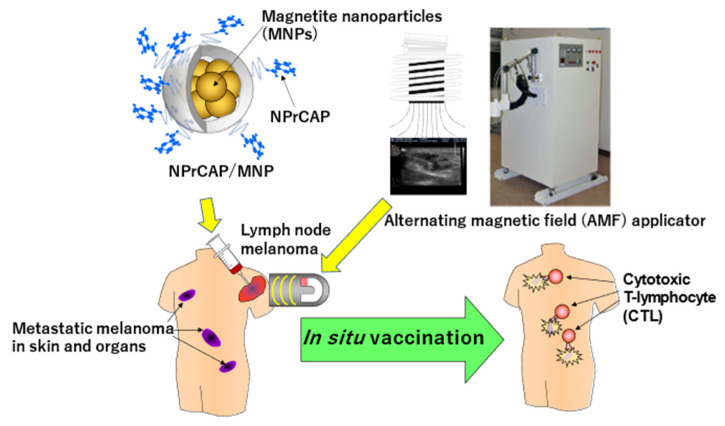
Flow of CTI therapy using conjugates of NPrCAP and MNP with exposure to alternating magnetic field (AMF) and growth inhibition of metastatic melanoma lesions by immunomodulated in situ vaccination.

Our approach is based on the combination of direct killing of melanoma cells, by chemotherapeutic and thermo-therapeutic effects of NPrCAP/MNPs with exposure to AMF, and indirect killing by immune reaction (in situ vaccination immunotherapy). This approach also provides a mechanism for producing a tumor-specific drug delivery system (DDS) that achieves selective melanoma cell death. This can then induce HSP production through either a necrotic or non-necrotic process or a combination of the two, without damaging non-cancerous tissues. This cascade can further generate an immune reaction targeted to other metastatic melanoma lesions, hence providing an “in situ vaccination” strategy” (Figure 19). There is, however, still a limitation in our current CTI therapy that should be resolved in prospective studies. This limitation is related to the patient selection and drug administration method. Currently, our NPrCAP/MNP needs to be given directly to regional lymph nodes or metastatic tumor lesions through ultrasound-guided needles (Figure 17). For such an approach, the ideal candidate patients would be those who have metastatic regional lymph nodes or superficial tumor lesions. In the future, however, this limitation will be resolved, because we have shown by electron microscopy [1] that *ip*-administered NPrCAP/MNPs are selectively transported into metastatic tumor lesions through melanogenesis-mediated selective DDS. There are no systemic adverse events except for the development of vitiligo-like depigmentation at distant, non-treated body sites, possibly through selective disintegration of melanocytes by in situ vaccination immune reaction (see DMC in Figure 10). In addition, we found that CTI therapy induced the infiltration of CD8^+^ T cells into melanoma tissue, indicating that this therapy converted a poorly immunogenic tumor to a highly immunogenic tumor. Therefore, our future studies will include efforts to further enhance the immune response to CTI therapy by combining it with current immunotherapeutic strategies such as ICIs.

## Abbreviations

AMF	alternating magnetic field
APTES	3-aminopropyltriethoxysilane
BSO	buthionine sulfoxide
CA-CysC	5-*S*-cysteaminyl-3-*S*-cysteinylcatechol
4-*S*-CAP	4-*S*-cysteaminylphenol
CDR3	third complementarity-determining region
CP	cysteinylphenol
CTI	chemo-thermo-immuno
CTL	cytotoxic T lymphocytes
DC	dendritic cell
DDS	drug delivery system
DNM	dextran nanomagnetite
DOPA	3,4-dihydroxyphenylalanine
GMP	good manufacturing practice
HPLC	high-performance liquid chromatography
HSP	heat shock protein
ICI	immune checkpoint inhibitor
IFN	interferon
MCL	magnetite cationic liposome
MEK	mitogen-activated protein kinase/extracellular receptor kinase
MHC	major histocompatibility complex
MITF	microphthalmia transcription factor
MNP	magnetite nanoparticle(s)
MSH	melanocyte-stimulating hormone
MTT	3-[4,5-dimethylthiazol-2-yl]-2,5-diphenyltetrazolium bromide
NAcCAP	*N*-acetyl-4-*S*-CAP
NPCMD	*N*-propionyl-4-*S*-cysteaminylphenol/maleimide-dextran
NPrCAP-SH	*N*-(1-mercaptopropionyl)-4-*S*-cysteaminyl phenol
NPrCAP/MNP	*N*-propionyl-4-*S*-cysteaminylphenol/magnetite nanoparticle
NPrCAP/PEG/MNP	*N*-propionyl-4-*S*-cysteaminylphenol/polyethylene glycol/magnetite nanoparticle
NPrCAP/PEG/APTES/DNM	*N*-propionyl-4-*S*-cysteaminylphenol/polyethylene glycol/3-aminopropyltriethoxysilane/dextran nanomagnetite
NPrCAQ	*N*-propionyl-4-*S*-cysteaminyl-1,2-benzoquinone
ROS	reactive oxygen species
TCR	T-cell receptor
TIL	tumor-infiltrating lymphocyte
TRIL	TNF-related apoptosis-inducing ligand
TRPs	tyrosinase related proteins
